# Low-dose clomiphene citrate does not reduce implantation and live birth rates in otherwise unstimulated modified natural cycle IVF—retrospective cohort study

**DOI:** 10.1007/s00404-022-06878-6

**Published:** 2022-12-16

**Authors:** Flavia Grädel, Michael von Wolff, Alexandra Sabrina Kohl Schwartz, Vera Ruth Mitter

**Affiliations:** 1 Division of Gynecological Endocrinology and Reproductive Medicine, Inselspital, University Women’s Hospital, University of Bern, Theodor-Kocher-Haus, Friedbühlstrasse 19, 3010 Bern, Switzerland; 2 Faculty of Medicine, University of Bern, Murtenstrasse 11, 3010 Bern, Switzerland; 3 Division of Reproductive Medicine and Gynaecological Endocrinology, Women’s Hospital, Cantonal Hospital Lucerne, Spitalstrasse, 6000 Lucerne, Switzerland

**Keywords:** Endometrium, Clinical pregnancy, Pregnancy rate, Modified natural-cycle, Bern IVF Cohort, FIVNAT

## Abstract

**Research question:**

Does antioestrogen effect of clomiphene citrate (CC) on the endometrium reduce implantation and thereby decrease pregnancy and live birth rate per transferred embryo?

**Methods:**

In this cohort, unstimulated IVF cycles modified with clomiphene citrate (CC-NC-IVF) and unstimulated, natural IVF cycles (NC-IVF) conducted between 2011 and 2016 were included. CC was applied in a dosage of 25mcg per day, starting on cycle day 7 until ovulation trigger day. Primary outcomes were clinical pregnancy rate, defined as amniotic sac visible in ultrasound, and live birth rate per transferred embryo. Miscarriage rate calculated as amniotic sac not ending in a live birth was secondary outcome. A modified mixed-effect Poisson regression model was applied, and adjustments were made for female age, parity, type and cause of infertility. Additionally, stratification by parity and age was performed.

**Results:**

Four hundred and ninety-nine couples underwent a total of 1042 IVF cycles, 453 being NC-IVF and 589 being CC-NC-IVF cycles. Baseline characteristics of both groups did not differ. Addition of CC did neither decrease clinical pregnancy rate (aRR 0.86; 95% CI 0.67–1.12) nor live birth rate per transferred embryo (aRR 0.84; 95% CI 0.62–1.13) in comparison with NC-IVF. Miscarriage rate did not differ between CC-NC-IVF and NC-IVF (aRR 0.95; 95% CI 0.57–1.57).

**Conclusion:**

Low-dose CC does not reduce pregnancy or live birth rate per transferred embryo. It can be used in infertility treatment without negatively affecting the endometrium and implantation.

**Supplementary Information:**

The online version contains supplementary material available at 10.1007/s00404-022-06878-6.

## What does this study add to the clinical work

**Table:** 

It is unclear if the antiestrogen effect of Clomiphen citrate reduces implantation after fresh embryo transfer. Low-dose CC (25 mcg) added to natural cycle IVF does not interfere with implantation and therefore not reduce pregnancy or live birth rate per transferred embryo.	

## Introduction

Clomiphene citrate (CC) is a selective oestrogen receptor modulator (SERM) that blocks the hypothalamic oestrogen receptors. This mimics a low serum concentration of oestrogen, which in turn induces negative feedback at the hypothalamic and pituitary glands, leading to increased secretion of gonadotropin-releasing hormone (GnRH) at the hypothalamus and of follicle-stimulating hormone (FSH) and luteinizing hormone (LH) from anterior pituitary. This induces follicular maturation in the ovaries, and the consecutive LH surge thereby eventually induces ovulation [1, 2].

In medically assisted reproduction (MAR), CC has been used for ovulation induction in case of dysovulatory cycles with the aim to increase the chance for natural conception—if necessary in combination with intrauterine insemination [3]. CC has also been added in doses of 25–100 mcg to unstimulated (CC-NC-IVF) and minimally stimulated in vitro fertilization (IVF) cycles in assisted reproductive technology (ART) treatments to increase the chance of successful oocyte gain at oocyte pick-up (OPU). It also reduces the risk for premature ovulation by impeding LH surge [4, 5]. CC is especially recommended for low-responders [6, 7] or is a low-cost alternative to regular IVF often performed in low-income countries [8].

Despite its benefits regarding successful OPU outcomes, CC has been suggested to interfere with successful embryo implantation. It reduces endometrial thickness due to its antiestrogenic activity [9], and it leads to a number of morphological irregularities in the endometrium such as increased stromal oedema and decreased glandular expression [10–12]. Reduced endometrial thickness is associated with lower chances for implantation and successful pregnancy outcome [13]. In CC-NC-IVF, CC is applied to improve oocyte gain but only a few studies assessed the impact on the endometrium and implantation by addressing clinical pregnancy rates and live births in comparison with unstimulated natural cycle IVF (NC-IVF). Those studies find mixed results with one study showing reduced implantation and pregnancy rates with CC [14], one not finding any differences [15] and one suggesting increased pregnancy rates with the addition of CC [16]. Results remain inconclusive and the studies differ in design, dosages of CC, and treatment schemes. It is further suspected that CC affects the function of fallopian tubes, increasing the risk for ectopic pregnancies [11, 17].

The aim of the present study is to assess the effect on endometrial receptivity of low-dose CC (25 mcg) used in CC-NC-IVF. Clinical pregnancy rates proving successful implantation, livebirth and miscarriage rates of embryos derived from CC-NC-IVF are compared to rates of embryos of NC-IVF.

## Materials and methods

### Data sources

**Fig. 1:**
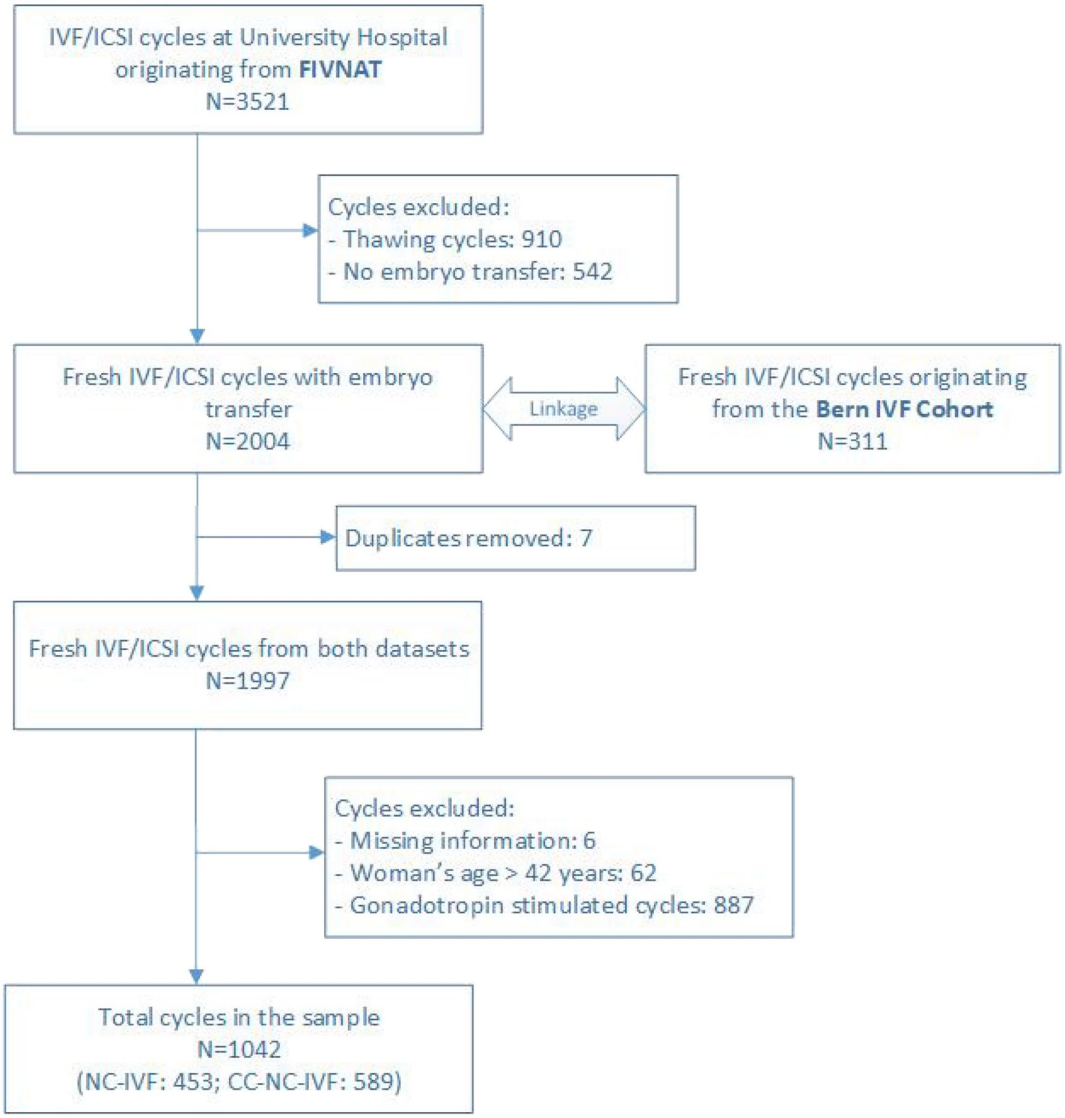
Data sources and creation of study population

Data for the present study were extracted from two different sources, the Swiss ART registry FIVNAT [18] and the Bern IVF Cohort [19]. FIVNAT provided data on all cycles performed at the University Hospital’s infertility centre between 2011 and 2016 (*n* = 3456). Thawing cycles (*n* = 910) and cycles without embryo transfer (*n* = 542) were excluded. The Bern IVF Cohort delivered data concerning all transfers of fresh embryos that led to a pregnancy (*n* = 311). Cycles with embryo transfer of FIVNAT (*n* = 2004) and Bern IVF Cohort (*n* = 311) were linked. Inconsistencies were clarified using medical records to increase data quality and to eliminate duplicates (Fig. 1).

### Study population

All NC-CC-IVF and all NC-IVF cycles performed between 2011 and 2016 at the Bern University Hospital were considered for the study if at least one fresh cleavage stage embryo was transferred (*n* = 1997). The following cycles were excluded: cycles with missing or inconsistent information (*n* = 6), maternal age over 42 years (*n* = 62) and cycles using additional gonadotropins for stimulation (*n* = 887). Finally, 1042 cycles were included, 453 NC-IVF cycles and 589 NC-CC-IVF cycles (Fig. 1). The ethics commission of the canton of Bern approved the study on February 26, 2020 (BASEC 2020-11800021).

### Treatment protocols

Treatment regime was a mutual decision between patient and physician. The decision was based on personal preferences regarding the different characteristics of NC-IVF and NC-CC-IVF and feasibility [20]. Both treatments required a regular menstrual cycle. CC was more likely to be administered in case of premature ovulation in a previous treatment cycle. In NC-IVF aspiration was scheduled when the diameter of the leading follicle was ≥ 16 mm and E2 concentration was ≥ 700 pmol/L. In CC-NC-IVF, patients took 25 mg CC in the morning, beginning on cycle day 7 until trigger day [15]. OPU was scheduled when diameter of the leading follicle reached ≥ 16 mm and E2 concentrations were ≥ 700L depending on the number of follicles. Ovulation was triggered with subcutaneous injection of 5000 IU of hCG 36–36.5 h before aspiration. OPU was performed without anaesthesia, using 19G monoluminal needles. Follicles were flushed 5 times as described elsewhere [21]. Oocytes were fertilized by intracytoplasmic sperm injection (ICSI) or rarely by IVF. All procedures were carried out in the same laboratory under standardized conditions. Embryo transfer of the available embryos (1–2) was performed at cleavage stage, 2–3 days after OPU. At that time, the vitrification of embryos was not allowed in Switzerland, which prohibited embryo selection. If both embryos kept in culture-reached cleavage stage, double embryo transfer was performed.

Luteal phase supplementation using intravaginal micronized progesterone 200 mg once a day was applied if necessary, oestrogen was never added [22]. At 14 days after OPU, beta-HCG was tested in serum, if positive an ultrasound scan was performed two weeks later. Clinical pregnancy was confirmed by sonographic detection of an amniotic sac.

### Statistical analysis

Primary outcomes were defined as number of amniotic sacs detected by ultrasound and the number of live births per embryo transferred. The secondary outcome was defined as the number of sonographically detected amniotic sacs not resulting in a live birth, therefore being classified as a miscarriage.

Baseline characteristics for each cycle of both treatment groups were analysed using linear regression for the comparison of continuous variables and logistic regression for the comparison of binary variables. For the comparison of the main indications for IVF treatment, a Chi-square test was applied. A modified multilevel Poisson regression model was used to calculate rate ratios of the primary outcomes [23]. This model accounts for the dependency of treatment cycles as the couples and the cycles define the levels. The number of embryos transferred within the same cycle is considered the count to start with and the amniotic sacs or the live births are the count outcome [24]. The same model was applied to assess the miscarriage rate where the number of amniotic sacs detected by ultrasound was the start count and the number of amniotic sacs lost was considered the count outcome [25].

The model was adjusted for the following co-variates: female age at the time of oocyte retrieval (continuous), parity (yes or no), type of infertility (primary or secondary) and main cause of infertility categorized as: reduced ovarian reserve, tubal factor, endometriosis rASRM stage I/II, endometriosis rASRM stage III/IV, anovulation, male factor, idiopathic or other reasons. A *p* value of < 0.05 was considered statistically significant.

All analyses were conducted in STATA Version 16 (StataCorp, College Station, Texas, USA).

### Sensitivity analysis

Several sensitivity analyses were conducted. The first including only one cycle per included women to avoid oversampling of couples with many cycles and potentially lower chances for treatment success. The second subanalysis included only couples, which did not switch treatment between NC-IVF and CC-NC-IVF during the inclusion period (*n* = 385). In the third analysis, the women were stratified by age (≤ 35; > 35) to assess the effect of age-related fertility decline [26]. Finally, the analysis was stratified by parity (nulliparous; parous) to assess the impact of parity as a positive predictive factor.

## Results

### Main analysis

In this study, 499 couples underwent a total of 1042 IVF cycles, 589 of them as CC-NC-IVF and 453 as NC-IVF. Of all couples, 239 (47.9%) contributed only one cycle to the sample and 260 (52.1%) contributed between 2 and 18 cycles. In CC-NC-IVF in 35.3% of cycles, more than one oocyte was collected, whereas in NC-IVF this was only the case in 4% of the cycles, leading to a higher proportion of double embryo transfers in CC-NC-IVF. In total, 1173 embryos were transferred. 711 embryos in CC-NC-IVF cycles with 467 (79.3%) in single and 244 (20.7% of cycles) in double transfers. 462 embryos in NC-IVF cycle with 444 (98%) being single and 18 (2% of cycles) being double embryo transfers (Table 1).

**Table 1:** Baseline characteristics of cycles by population

	NC-IVF	CC-NC-IVF	*p* value
	(*n* = 453)	(*n* = 589)	
Maternal age at oocyte pick-up (years)			0.498
Mean (SD)	35.9 (3.9)	36.0 (4.0)	
Duration of infertility (years)			0.567
Mean (SD)	4.1 (2.3)	4.0 (2.0)	
Maternal age at oocyte pick-up (group)			0.180
20–24	0 (0.0%)	3 (0.5%)	
25–29	35 (7.7%)	38 (6.5%)	
30–34	119 (26.3%)	163 (27.7%)	
35–39	217 (47.9%)	255 (43.3%)	
40–42	82 (18.1%)	130 (22.1%)	
Duration of infertility (group)			0.859
< 1 Year	4 (0.9%)	5 (0.8%)	
1–2 Years	114 (25.2%)	139 (23.6%)	
3–5 Years	240 (53.0%)	328 (55.7%)	
> 5 Years	95 (21.0%)	117 (19.9%)	
Primary or secondary infertility			0.029
Primary	343 (75.7%)	410 (69.6%)	
Secondary	110 (24.3%)	179 (30.4%)	
Main indication for IVF treatment			0.804
Ovarian reserve	11 (2.4%)	16 (2.7%)	
Tubal factor	49 (10.8%)	76 (12.9%)	
Endometriosis rASRM I/II	42 (9.3%)	51 (8.7%)	
Endometriosis rASRM III/IV	12 (2.6%)	18 (3.1%)	
Anovulation	4 (0.9%)	5 (0.8%)	
Male factor	244 (53.9%)	328 (55.7%)	
Idiopathic	88 (19.4%)	91 (15.4%)	
Other	3 (0.7%)	4 (0.7%)	
Maternal parity at this cycle			0.134
Nulliparous	425 (93.8%)	538 (91.3%)	
Parous	28 (6.2%)	51 (8.7%)	
Oocytes collected at oocyte pick-up			< 0.001
1	435 (96.0%)	381 (64.7%)	
2	18 (4.0%)	179 (30.4%)	
3	0 (0.0%)	29 (4.9%)	
Number of embryos transferred			< 0.001
Single embryo transfer	444 (98.0%)	467 (79.3%)	
Double embryo transfer	9 (2.0%)	122 (20.7%)	
Number of children born			0.258
No child	393 (86.8%)	516 (87.6%)	
1 child	60 (13.2%)	70 (11.9%)	
2 children	0 (0.0%)	3 (0.5%)	

*N* number, *SD* standard deviation, *rASRM* revised American society of reproductive medicine classification for endometriosis

The baseline characteristics for both groups were not different regarding female age and infertility characteristics. However, the number of oocytes retrieved, and the number of double embryo transfers was higher in CC-NC-IVF cycles (Table 1).

The results per cycle while ignoring the number of embryos transferred were the following:

In CC-NC-IVF, 96 (16.3%) cycles resulted in a clinical pregnancy of which 73 (76%) (70 singletons and 3 twins; 12.39%) ended in a live birth and 23 (24.0%) in a miscarriage, 19 of those miscarried (19.8%) in the first trimester, one in the second and three cycles ended with ectopic pregnancies. In NC-IVF, 80 (17.6%) cycles resulted in a clinical pregnancy of which 60 (75%) ended in a livebirth of a singleton and 20 (25%) in a miscarriage; 18 (22.5%) within the first trimester and 2 in the second.

The unadjusted analysis revealed that the addition of CC did not decrease the clinical pregnancy rate (rate ratio (RR) 0.84; 95% CI 0.63–1.11; *p* = 0.21) or live birth rate per transferred embryo (RR 0.82; 95% CI 0.59–1.14; *p* = 0.24). Miscarriage rate was not different in CC-NC-IVF compared to NC-IVF (RR 1.14; 95% CI 0.66–1.98; *p* = 0.64) either. The adjusted analysis confirmed these results showing an aRR of 0.86 for clinical pregnancy (95% CI 0.67–1.12; *p* = 0.27) and 0.84 for live birth per transferred embryo (95% CI 0.62–1.13; *p* = 0.25). Adjustments did not reveal any differences in misscariage rates between CC-NC-IVF compared to NC-IVF either (aRR 0.95; 95% CI 0.57–1.60; *p* = 0.86) (Tables 2, 3, 4).

**Table 2:** Clinical pregnancy rate per transferred embryo

Stimulation protocol	RR	*p*	95% CI
NC-IVF	1.00 (Ref)		
CC-NC-IVF (crude)	0.84	0.21	0.63–1.11
CC-NC-IVF (adjusted)^a^	0.86	0.27	0.67–1.12

*RR* rate ratio, *p p* value, *95% CI* 95% confidence interval, *NC-IVF* unstimulated, natural cycle in vitro fertilization, *CC-NC-IVF* clomiphene-stimulated IVF

^a^Adjusted for female age, parity, duration of infertility, primary/secondary infertility, cause of infertility

**Table 3:** Live birth rate per transferred embryo

Stimulation protocol	RR	*p*	95% CI
NC-IVF	1.00 (Ref)		
CC-NC-IVF (crude)	0.82	0.25	0.59–1.14
CC-NC-IVF (adjusted)^a^	0.84	0.24	0.62–1.13

*RR* rate ratio, *p p* value, *95% CI* 95% confidence interval, *NC-IVF* unstimulated, natural cycle in vitro fertilization, *CC-NC-IVF* clomiphene-stimulated IVF

^a^Adjusted for female age, parity, duration of infertility, primary/secondary infertility, cause of infertility

**Table 4:** Miscarriages rate per amniotic sac

Stimulation protocol	RR	*p*	95% CI
NC-IVF	1.00 (Ref)		
CC-NC-IVF (crude)	1.14	0.64	0.66–1.98
CC-NC-IVF (adjusted)^a^	0.95	0.86	0.57–1.60

*RR* rate ratio, *p p* value, *95% CI* 95% confidence interval, *NC-IVF* unstimulated, natural cycle in vitro fertilization, *CC-NC-IVF* clomiphene-stimulated IVF

^a^Adjusted for female age, parity, duration of subfertility, primary/secondary infertility, cause of infertility

### Sensitivity analyses

If exclusively the first or only cycle of each couple was included in the analysis, 210 had CC-NC-IVF and 289 had NC-IVF treatment. The analysis of this subsample revealed no differences between the two treatment types. Of the couples that contributed more than one cycle (*n* = 260), most couples had the same treatment (*n* = 146; 56.2%) in every included cycle, whereas 114 (43.8%) did switch and had cycles of both treatments. In total, 385 couples contributed one or all cycles of the same treatment, which resulted in 654 cycles analysed in this subanalysis. Results of modified Poisson regression including only couples continuing with the same treatment did not show differences between CC-NC-IVF and NC-IVF either. Finally, age did not reveal any differences between CC-NC-IVF and NC-IVF, whereas parous women seemed to have a better clinical pregnancy rate in NC-IVF treatment, but this was not seen for live births (Online Resource 1).

## Discussion

This study suggests that low-dose CC, such as 25mcg in modified CC-NC-IVF, does not have a clinically relevant impact on endometrial receptivity and consecutively on implantation when compared to unstimulated NC-IVF. This result was robust in different sensitivity analyses.

In employing a woman’s physiological menstrual cycle, NC-IVF offers an ideal model to analyse the clinical impact of the known unfavourable effects of CC on endometrium and reproductive function. Because of the prohibition of embryo selection in Switzerland, the potential bias introduced by embryo selection is not present. An additional strength of the present study is the focus on one IVF centre with large experience and standardized laboratory protocols. To increase power, all available cycles and thus more than one cycle per couple were included. The hierarchical count model accounts for the dependency of the cycles within the same couple and for the embryos transferred in the same cycle, and several sensitivity analyses were conducted to assess potential influence of further factors. Limitations are found in the study’s observational design and the non-randomized allocation of IVF treatment according to patients and physicians’ preference, which can lead to selection bias. Additionally, data might be skewed by the fact that women who got pregnant in their first treatment cycle dropped out faster and contributed fewer, but successful cycles. The study does not provide information on earlier ART cycle failure or OPU outcome, where CC has been shown to have a positive influence, mainly in avoiding preterm ovulation [15].

To our knowledge, only few previous studies assessed pregnancy outcome after CC-NC-IVF in comparison with NC-IVF and it is not possible to draw clear conclusions. In a prospective trial by Ingerslev et al. 132 women with no previous IVF attempts were randomized to either 100 mg CC on cycle days 3–7 (*n* = 68 with 111 cycles) or to no stimulation at all (*n* = 64 with 114 cycles). More cycles with successful embryo transfers were seen in the CC group (*n* = 59 vs *n* = 29). In addition, a higher proportion of clinical pregnancies per transfer was achieved in the CC group (33.9% vs 13.8%, *p* = 0.047), and the difference was even larger as per cycle started (18.0% vs 3.5%, *p* < 0.001). The higher embryo transfer rate and success by cycle is believed to be related to the positive impact of CC on OPU success. Comparison to the present study is, however, limited by the fact that a dose of 100 mcg CC would have a different effect on the endometrium than the minimal dose of 25 mcg applied. No results for miscarriages or livebirths were presented, and the results were not corrected for dependency of cycles in the same women nor for the number of embryos transferred [16].

Abe et al. assessed pregnancy rates of NC-CC-IVF in 834 women following different types of embryo transfers (fresh cleavage, blastocyst or a frozen cleavage or blastocyst). Following the transfer of a single fresh cleavage embryo, the only setting comparable to the one in the present study, in 24.0% of cycles, a clinical pregnancy was detected and in 18.7% of the cycles, a livebirth was achieved. These results and the miscarriage risk were highly dependent on age of the women [5].

In a cohort study by Kato et al. including only one cycle per women, pregnancy success following CC-NC-IVF (*n* = 24) was compared to NC-IVF (*n* = 157). For stimulation, 50–100 mg CC on day 3 until the day before ovulation trigger was applied. In contrast to Ingerslev et al. clinical pregnancy (45.8% vs 69.4%) and livebirth rates (29.2% vs 56.1%) were reported to be significantly lower per single blastocyst transfer in CC-NC-IVF. The adverse effect of CC was not seen after the transfer of a vitrified embryo, suggesting frozen embryo transfer within natural cycle being a better option following CC stimulation, a fact confirming the hypothesis of a negative effect of CC on the endometrium and not on follicular maturation. However, the number of fresh transfers following CC-NC-IVF was very low [14].

Von Wolff et al. compared NC-IVF and CC-NC-IVF cycles stimulated with 25 mg CC on day 7 until the day of ovulation trigger in 112 women having both treatments each. In contrast to the previously discussed studies, they did not find any differences in pregnancy and implantation rates compared to NC-IVF, but the primary outcome of the study was preterm ovulation and the sample size was limited [15]. The present study could confirm these results with a larger sample size, including the cycles from the previous study, referring to 20.2% of all cycles.

A recent study found a CC-induced delay of endometrial maturation in women treated with 100mcg CC for 5 days [27]. Endometrial thickness can be reduced by CC by around 1–2 mm [9, 28, 29]. Thin endometrium has been shown to decrease pregnancy success in gonadotropin-stimulated [13] as well as in gonadotropin-unstimulated cycles [30]. Additionally, an association of endometrial thickness at trigger day with lower ongoing pregnancy rates has been explicitly shown for minimal stimulated IVF cycles with added CC [31]. However, it remains unclear whether the endometrium thinning effect is the reason for lower pregnancy rates in CC treatments compared to other ART [32]. Further negative effects of CC on the reproductive system have previously been postulated: In rats, CC treatment induced cell type specific apoptosis in the uterus and in the fallopian tube, changed the uterine morphologic conditions and decreased the expression of oestrogen receptor-alpha through its activation. All these changes may interfere with implantation and pregnancy success and the exact biological mechanisms, and their clinical relevance in humans has not been entirely clarified [11, 33]. A higher ectopic pregnancy rate as a result of CC cycles in humans has already been shown by several studies. A total of three ectopic pregnancies out of 96 clinical pregnancies (3.1%) were observed in the present study, which is high compared to a presented 1.4% in pregnancies following ART overall [17, 34].

The heterogenic outcomes of previous studies can hardly be explained. It is possible that the unfavourable molecular impacts of CC on endometrial function are dose dependent or mainly associated with long-term or chronic use of CC. However, an older trial did not find dose-dependent effects of CC [35].

In the present study, differences in implantation, miscarriage and live birth rates between NC-IVF and CC-NC-IVF were not identified. It suggests that CC at the dose of 25mcg does not have a clinically relevant impact on endometrial function affecting implantation, miscarriage or live birth rates. Low-dose CC is a low-cost alternative that can be given until the day of ovulation trigger in fresh IVF cycles. However, more evidence of randomized clinical trials comparing unstimulated and CC stimulated IVF cycles involving different CC dosages are required to define cut-offs of save CC dosages.

## Supplementary Information

Below is the link to the electronic supplementary material.

Supplementary file1 (DOCX 17 KB)

## Data Availability

The participants did not give written consent for their data to be shared publicly, so due to the sensitive nature of the research supporting data is not available. Data of Swiss IVF Registry is subject to third party restrictions. More information on FIVNAT can be found on www.sgrm.org.
