# Supplementary material for: Low-dose clomiphene citrate does not reduce implantation and live birth rates in otherwise unstimulated modified natural cycle IVF—retrospective cohort study
Source: Arch Gynecol Obstet. 2022 Dec 16;307(4):1073–81. doi: 10.1007/s00404-022-06878-6 (PMC10023627; doi:10.1007/s00404-022-06878-6)
Supplement: Supplementary file 1 — Supplementary file1 (DOCX 17 KB) [file 404_2022_6878_MOESM1_ESM.docx]

Online resource 1 Results of different sensitivity analyses

|  | Age at OPU  ≤ 35 years | Age at OPU  > 35 years | Parity  nulliparous | Parity  parous | First or only cycle | Same treatment* |
| --- | --- | --- | --- | --- | --- | --- |
|  | RR (95%CI) | RR (95%CI) | RR (95%CI) | RR (95%CI) | RR (95%CI) | RR (95%CI) |
| n cycles | n=358 | n=684 | n=963 | n=79 | n=499 | n=654 |
| Clinical pregnancy | | | | | | |
| NC-IVF | 1.00 (Ref) | 1.00 (Ref) | 1.00 (Ref) | 1.00 (Ref) | 1.00 (Ref) | 1.00 (Ref) |
| CC-NC-IVF crude | 0.73 (0.51-1.04) | 0.96 (0.64-1.43) | 0.91 (0.67-1.25) | 0.51 (0.31-0.84) | 0.91 (0.63-1.31) | 0.74 (0.54-1.01) |
| CC-NC-IVF  adjusted^a^ | 0.76 (0.54-1.07) | 0.84 (0.56-1.28) | 0.94 (0.70-1.27) | 0.56 (0.33-0.94) | 1.07 (0.75-1.52) | 0.82 (0.61-1.11) |
| Live birth | | | | | | |
| NC-IVF | 1.00 (Ref) | 1.00 (Ref) | 1.00 (Ref) | 1.00 (Ref) | 1.00 (Ref) | 1.00 (Ref) |
| CC-NC-IVF crude | 0.79 (0.54-1.15) | 0.86 (0.50-1.47) | 0.87 (0.59-1.26) | 0.57 (0.34-0.96) | 0.92 (0.61-1.40) | 0.67 (0.47-0.97) |
| CC-NC-IVF  adjusted^a^ | 0.81 (0.56-1.17) | 0.71 (0.42-1.20) | 0.90 (0.63-1.29) | 0.60 (0.35-1.03) | 1.08 (0.71-1.64) | 0.75 (0.53-1.07) |

^a^ adjusted for female age, parity, duration of subfertility, primary/ secondary infertility, cause of infertility

Abb.: RR: rate ratio, 95% CI: 95% confidence interval; NC-IVF: unstimulated, natural cycle in vitro fertilization; CC-NC-IVF: clomiphene-stimulated IVF; n: number of cycles
